# Screening novel antiviral compounds to treat *Clostridioides difficile* infections

**DOI:** 10.1371/journal.pone.0309624

**Published:** 2024-12-13

**Authors:** Brice J. Stolz, Ahmed A. Abouelkhair, Mohamed N. Seleem

**Affiliations:** 1 Department of Biomedical Sciences and Pathobiology, Virginia-Maryland College of Veterinary Medicine, Virginia Polytechnic Institute and State University, Blacksburg, Virginia, United States of America; 2 Center for One Health Research, Virginia Polytechnic Institute and State University, Blacksburg, Virginia, United States of America; Defense Threat Reduction Agency, UNITED STATES OF AMERICA

## Abstract

*Clostridioides difficile* is a major cause of nosocomial infections, often associated with individuals who have gut dysbiosis from previous antibiotic therapies. *C*. *difficile* infections (CDI) have a high recurrence rate and impose significant financial and mortality burdens on the healthcare system. Therefore, novel anti-*C*. *difficile* drugs are urgently needed to treat and reduce the severity and recurrence of infection. In this study, we screened a library of 618 antiviral drugs to identify a potential candidate for repurposing as novel anti-*C*. *difficile* therapeutics. Following our preliminary screening, we identified 9 novel compounds that inhibited *C*. *difficile* at a concentration of 16 μM or lower. Among these, 4 antiviral compounds demonstrated the most potent anti-*C*. *difficile* activity against a panel of 15 *C*. *difficile* isolates, with minimum inhibitory concentrations (MICs) comparable to the drug of choice, vancomycin. These include rottlerin (MIC_50_ = 0.25 μg/mL), α-mangostin (MIC_50_ = 1 μg/mL), dryocrassin ABBA (MIC_50_ = 1 μg/mL), and obefazimod (MIC_50_ = 4 μg/mL). All exhibited minimal to no activity against representative members of the human gut microbiota. Interestingly, α-mangostin, a natural xanthone derived from the mangosteen fruit, exhibited strong bactericidal action, clearing a high inoculum of *C*. *difficile* in less than an hour. All other drugs exhibited bacteriostatic activity. Given their characteristics, these compounds show great promise as novel treatments for CDI.

## Introduction

Healthcare-associated infections are one of the greatest burdens on healthcare systems. Most bacteria responsible for a part of this burden are associated with antimicrobial resistance, with one notable exception: *C*. *difficile*. Despite lacking a significant antimicrobial resistance profile, *C*. *difficile* is still considered an urgent threat and one of the deadliest bacterial infections responsible for approximately 30,000 deaths annually, primarily affecting older populations [1–5]. The incidence of *C*. *difficile* infections (CDI) in the United States has increased over the last two decades and has remained relatively stable from 2021 to 2022, with the COVID-19 pandemic seeing a drop in cases due to improved strategies preventing the spread of microorganisms [2, 3, 6]. Typically, *C*. *difficile* targets patients receiving antibiotic-based therapy. This can be attributed to that the use of antibiotics leads to gut dysbiosis, which increases the growth and colonization of *C*. *difficile* in the gut. As a consequence, colonizing *C*. *difficile* produces toxins that can cause damage of the tight junctions of the intestinal epithelium eliciting a variety of symptoms, ranging from mild diarrhea to intestinal tissue necrosis and pseudomembranous colitis [7–9].

Novel antibiotic therapies approved for CDI have been conspicuously lacking. In the past, the recommended drugs for mild to severe CDI were metronidazole and vancomycin, respectively. Fidaxomicin is the most recently approved new antibiotic for CDI, having been approved nearly 40 years ago. Due to the high rates of treatment failure and recurrence, metronidazole is no longer recommended; and vancomycin and fidaxomicin are now the main therapeutic options for CDI treatments [8, 10, 11]. Despite the potency of these antibiotics against *C*. *difficile*, recurrence rates can still be significant even if they are an improvement over metronidazole. Fidaxomicin and vancomycin have both encountered recurrence rates of 20% or higher, with subsequently increasing chances of recurrence after the first episode [12, 13]. Hence, there is an immediate and growing need for new antibiotics to address the shortcomings of the existing therapeutics and their impact on the healthcare system.

Antivirals are one of the most prolific and growing categories of treatments since the first was approved in 1963 [14]. With the advent of COVID-19, antiviral research has become an area of increasing interest, and with it many antiviral compounds were approved for treatment or held back due to their pharmacokinetics [15]. Poor oral bioavailability is a challenge for many antivirals, which prevents them from being approved as new antivirals [16]. This might be advantageous for treating CDI, which requires drugs with low intestinal absorption to remain in the gut for a longer period of time [17–19]. Despite the need for more potent drugs for treating CDI with minimal intestinal absorption, screening antiviral drugs for possible activity against *C*. *difficile* has not been thoroughly investigated. Prior screens and clinical studies have demonstrated the potential of antiviral drugs, like nitazoxanide, a broad-spectrum antiviral and antiprotozoal drug, against *C*. *difficile* in comparison to vancomycin [20–22]. Moreover, antiviral compounds have been effective against other microorganisms. For instance, the antiviral hypericin increased the effectiveness of beta-lactam antibiotics against methicillin-resistant Staphylococcus aureus (MRSA) [23].

Given the potential of the aforementioned medications, the goal of this work is to screen an antiviral library to find antivirals with considerable potency against *C*. *difficile*. Screening an antiviral library revealed four antiviral compounds that had strong anticlostridial activity. These hits were assessed for potency and specificity against a panel of clinical isolates of *C*. *difficile* and the human gut microbiota, respectively. Antivirals also had their killing kinetics assessed to determine their killing kinetics over a 48-hour period.

## Materials and methods

### Bacterial strains & reagents

Bacterial strains utilized in this study were sourced from the Centers for Disease Control and Prevention (CDC, Atlanta, GA), Biodefense & Emerging Infections Research Resources Repository (BEI Resources, Manassas, VA) and the American Type Culture Collection (ATCC, Manassas, VA). Phosphate-buffered saline (PBS) (Corning, NY), Brain heart infusion broth (BHI) and De Man—Rogosa—Sharpe broth (MRS) (Becton, Dickson and Company, Franklin Lakes, NJ), yeast extract (Fisher Scientific Global Solutions, Suwanee, GA), L-cystine (Thermo Fisher Scientific, Waltham, MA), and vitamin K1, resazurin, and hemin (Sigma-Aldrich, St. Louis, MO) were purchased commercially.

### Compounds and libraries

The MCE antivirals library (Cat. No. HY-L027), which includes 618 unique compounds displaying antiviral activity, was purchased from MedChemExpress (Monmouth Junction, NJ). After an initial screening and confirmation, the active hits were purchased commercially as follows: rottlerin and dryocrassin ABBA (MedChemExpress; Monmouth Junction, NJ), *α*-mangostin (TargetMol Chemicals; Wellesley Hills, MA), and obefazimod (Ambeed; Arlington, IL). Vancomycin hydrochloride (Gold Biotechnology, Olivette, MO, USA) was included as a positive control.

### Screening assay against *C*. *difficile*

To discover antivirals with anti-*C*. *difficile* action, the MCE antivirals library was screened using the broth microdilution technique, as previously described [24–27], against *C*. *difficile* ATCC BAA 1870 at a fixed concentration of 16 μM. In brief, bacteria were streaked and grown anaerobically at 37°C for 48 hours on brain heart infusion supplemented (BHIS) agar plates. A 0.5 McFarland solution of *C*. *difficile* was then prepared and diluted in BHIS to reach an inoculum of ~5 × 10^5^ CFU/mL and placed in 96-well plates. The antiviral compounds were added at a concentration of 16 μM. The plates were then incubated anaerobically at 37°C for 48 hours. Using a BioTek Synergy H1 Microplate Reader with BioTek Gen 5 and Imager Software, the OD_600_ was determined. The compounds that inhibited >80% of the bacterial growth, as compared to the OD_600_ of the growth control wells (DMSO), were recognized as potential hits of interest and purchased commercially for further confirmation. GraphPad Prism version 10 was employed to illustrate the growth inhibition.

### Anticlostridial activity of the potent hits

The broth microdilution technique was used to test the antiviral hits against *C*. *difficile* ATCC BAA 1870 to identify the minimum inhibitory concentrations (MICs) of the promising hits [28–30]. The hits and the control antibiotic, vancomycin, were serially diluted along the 96-well plates. A bacterial solution equivalent to 0.5 McFarland standard was diluted in BHIS broth to obtain a final bacterial concentration of 5 × 10^5^ CFU/mL and added to the plates. These plates were then incubated anaerobically at 37°C for 48 hours. The lowest concentration at which agents could totally prevent bacterial growth was identified as the MIC. We selected hits with MIC ≤4μg/mL (rottlerin, dryocrassin ABBA, α-mangostin, and obefazimod) for further investigation. These four compounds were purchased commercially and examined against a panel of 15 clinical isolates of *C*. *difficile* in comparison to the control antibiotic, vancomycin. The test agent concentrations that inhibited 50% and 90% of the strains (MIC_50_ and MIC_90_, respectively), were determined. MICs were performed at least in two independent experiments, each containing the biological replicates.

### Antibacterial activity of potent hits against representative members of gut microbiota

The most potent hits’ MICs against gut microbiota strains were ascertained in accordance with previous studies [31–33]. To achieve a final bacterial concentration of 5 × 10^5^ CFU/mL, a 0.5 McFarland bacterial solution was diluted in BHIS broth (for *Bifidobacterium* and *Bacteroides* strains), and MRS broth (for *Lactobacillus*). After serially diluting this suspension with the hits and control antibiotics, it was incubated for 48 hours at 37°C in anaerobic conditions for *Bifidobacterium* and *Bacteroides* or in the presence of 5% CO_2_ for *Lactobacillus* before determining the MICs of the hits.

### Time-kill kinetics assay against *C*. *difficile*

In a time-kill experiment, potential antiviral drugs were challenged against *C*. *difficile* to ascertain the bactericidal or bacteriostatic nature of inhibition, as previously described [24, 34]. *C*. *difficile* ATCC 43255 and *C*. *difficile* ATCC BAA-1870 were grown overnight, then diluted 1:150 into sterile BHIS, yielding a concentration of approximately 5 × 10^5^ CFU/mL. *C*. *difficile* was treated with the hits and the control antibiotic, vancomycin, at a concentration of 5× MIC, and then cultured anaerobically at 37°C. DMSO was also included as an untreated control. At 0, 2, 4, 8, 12, 24, and 48 hours, aliquots were collected from each treatment, diluted, and plated onto pre-reduced BHIS agar plates. Plates were incubated in anaerobic conditions at 37°C before determining the bacterial CFU. A drug was deemed to be bactericidal if it reduced the initial inoculum by ≥3 log_10_ CFU/mL.

## Results

### Screening of the antiviral library against *C*. *difficile*

**Fig 1:**
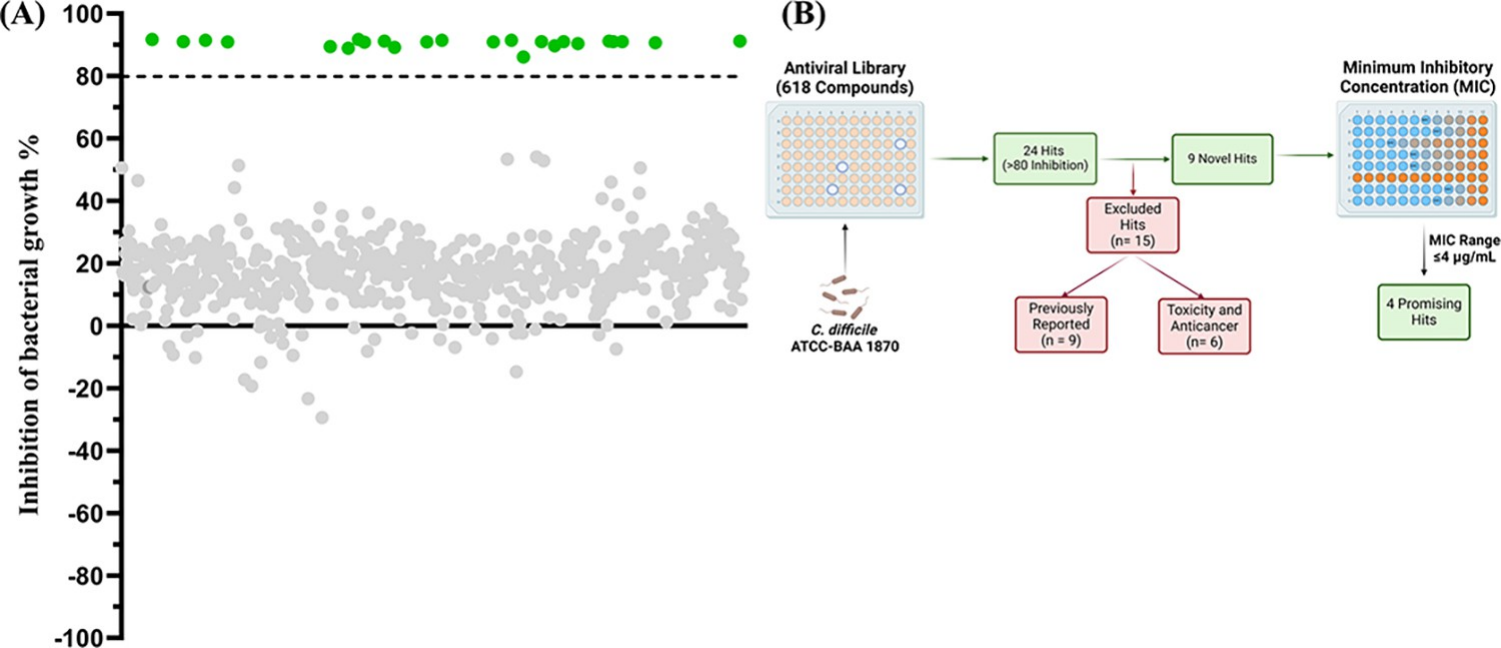
MCE antiviral library screening of 618 antivirals at 16 μM against *C*. *difficile* ATCC BAA-1870. Compounds with a greater than 80% inhibition of bacterial growth were identified as hits (highlighted in green), while hits below 80% inhibition were excluded due to lack of activity (in gray). 24 total hits were identified with 9 novel hits and 15 excluded hits. The 9 novel hits are: rottlerin, dryocrassin ABBA, α-mangostin, obefazimod, baloxavir, 4’-O-methylbavachalcone, TMC647055 (choline salt), brefeldin A, and maslinic acid. The 15 excluded hits can be found in S1 Table in S1 File.

To discover novel anti-*C*. *difficile* drugs, 618 antivirals (MCE antiviral library) were tested for possible anti-*C*. *difficile* action against the hypervirulent strain of *C*. *difficile* ATCC BAA-1870. The screening was done at an initial concentration of 16 μM using the broth microdilution method. A total of 24 compounds were found to possess anticlostridial action, effectively preventing the growth of *C*. *difficile* at the screening concentration (**Fig 1, Table 1, and S1 Table in S1 File**). Based on past literature, 15 hits that have previously been reported or recognized as unsafe or anticancer drugs [27, 28, 35–43] have been excluded (**S1 Table in S1 File**) [44–48]. The other unique 9 hits have been confirmed against *C*. *difficile*, and their MICs were determined. Out of these 9 hits, 4 antiviral compounds (rottlerin, α-mangostin, dryocrassin ABBA, and obefazimod) displayed the most potent activity against *C*. *difficile* ATCC BAA-1870 with MIC values of ≤4 μM (**Fig 1**). Due to their potency and lack of prior research, these promising hits were chosen for further investigation. Among the most promising hits in the library (**Table 1**), 3 were natural products (rottlerin, α-mangostin and dryocrassin ABBA), and 1 (obefazimod) is currently in clinical trials for HIV and ulcerative colitis treatments [49, 50].

**Table 1:** Description and MICs (μM) of potent antivirals against *C*. *difficile* ATCC BAA-1870.

No.	Compound ID	Description	MICs (μM)
**1**	Rottlerin	Natural product isolated from *Mallotus phillippinesis* and PKC-δ Inhibitor	0.5
**2**	Dryocrassin ABBA	Natural Product isolated from *Dryopteris crassirhizoma*	2
**3**	α-Mangostin	Natural Product isolated from *Garcinia mangostana*	4
**4**	Obefazimod	Anti-HIV & Colitis	4
**5**	Baloxavir	Antiviral	8
**6**	4’-O-Methylbavachalcone	Antiviral	8
**7**	TMC647055 (choline salt)	Antiviral	16
**8**	Brefeldin A	Antiviral lactone produced by fungi	16
**9**	Maslinic acid	Natural product isolated from *Olea europaea*	16

### Anticlostridial activity of the potent hits

The anticlostridial activity of the potent 4 hits was further confirmed by testing them against 15 clinical isolates of *C*. *difficile* (**Table 2**) and calculating the MIC_50_ and MIC_90_ of each drug. Against the tested strains of *C*. *difficile*, the activity of rottlerin (MIC_50_ = 0.25 μg/mL and MIC_90_ = 0.5 μg/mL) and α-mangostin (MIC_50_ = 1 μg/mL and MIC_90_ = 2 μg/mL) was comparable to those of vancomycin (MIC_50_ = 0.5 μg/mL and MIC_90_ = 1 μg/mL). Likewise, dryocrassin ABBA (MIC_50_ = 1 μg/mL and MIC_90_ = 4 μg/mL) had similar activity to vancomycin with a 2-fold difference in its MIC_90_. Conversely, obefazimod needed a 3- fold greater concentration to inhibit *C*. *difficile* (MIC_50_ = 4 μg/mL and MIC_90_ = 8 μg/mL) in comparison to vancomycin.

**Table 2:** MIC values of the most potent antivirals against clinical isolates of *C*. *difficile*.

*C*. *difficile* strain ID	MIC (μg/mL)				
	Antiviral hits				Control antibiotic
	Rottlerin	α-Mangostin	Dryocrassin ABBA	Obefazimod	Vancomycin
**ATCC-BAA 1870**	0.5	1	2	4	1
**ATCC 1871**	0.5	2	4	8	2
**ATCC 43255**	0.25	0.5	0.5	2	0.5
**ATCC 43598**	0.5	1	1	8	0.5
**BAA 630**	0.25	0.5	1	4	0.5
**NR-49288**	0.5	2	4	8	1
**NR-49302**	0.5	0.5	2	4	0.5
**NR-49308**	0.25	0.5	0.5	4	0.5
**NR-49313**	0.25	1	0.5	2	0.5
**NR-49319**	0.5	1	1	4	0.25
**CD-12**	0.25	1	0.5	4	1
**CD-16**	0.25	0.5	1	2	0.5
**CD-20**	0.25	0.5	1	4	0.5
**CD-21**	0.5	2	4	8	1
**CD-28**	0.25	1	1	8	1
**MIC**_**50**_	**0.25**	**1**	**1**	**4**	**0.5**
**MIC**_**90**_	**0.5**	**2**	**4**	**8**	**1**

### Antibacterial activity of the most potent hits against representative members of the human gut microbiota

To test the activity against gut microflora, selected antiviral compounds were screened against a panel of representative commensal gut microbiota including *Lactobacillus*, *Bacteroides*, and *Bifidobacterium*, starting at a concentration of 256 μg/mL. The selected hits and vancomycin had little to no effect on *Lactobacillus* strains apart from fidaxomicin (MIC values of 8 and 32 μg/mL against *L*. *rhamnosus* and *L*. *brevis* strains, respectively) (**Table 3**).

**Table 3:** MIC (μg/mL) values of selected antivirals and control antibiotics against representative members of human normal gut microbiota.

Bacterial strain ID	Minimum inhibitory concentration (μg/mL)				
	Antiviral hits				Control antibiotics
	Rottlerin	Obefazimod	α-Mangostin	Dryocrassin ABBA	Vancomycin
***L*. *brevis* 14864**	>256	>256	>256	>256	>256
***L*. *rhamnosus* 53103**	128	>256	>256	>256	>256
***L*. *casei* ATCC 334**	128	>256	>256	>256	>256
***B*. *fragilis* HM-709**	32	8	≤2	32	16
***B*. *fragilis* HM-714**	64	8	≤2	64	32
***B*. *fragilis* HM-710**	16	128	≤2	32	16
***B*. *adolescentis* HM-633**	≤2	>256	2	8	≤2
***B*. *breve* HM-411**	8	8	≤2	8	≤2
***B*. *breve* HM-1120**	4	8	≤2	8	≤2

Rottlerin, obefazimod, and dryocrassin ABBA showed activity at higher concentrations (MIC values ranged from 8 to 128 μg/mL) against *Bacteroides fragilis* strains in comparison to vancomycin (MIC values, 16–32 μg/mL); however, α-mangostin displayed potent activity at lower concentrations (MIC values ≤2 μg/mL) (**Table 3**).

Rottlerin, obefazimod, and dryocrassin ABBA showed activity at lower concentrations (MIC values, 4–8 μg/mL) against *Bifidobacterium breve* strains, whilst α-mangostin showed activity at lower concentrations (MIC values, ≤2 μg/mL) similar to the control antibiotic, vancomycin (**Table 3**). Overall, antiviral compounds showed similar or less activity against gut microflora when compared to drugs of choice for CDI, with the greatest difference in activity being against *Bifidobacterium* strains being at least 2–4 folds less active.

### Time-kill kinetics assay against *C*. *difficile*

**Fig 2:**
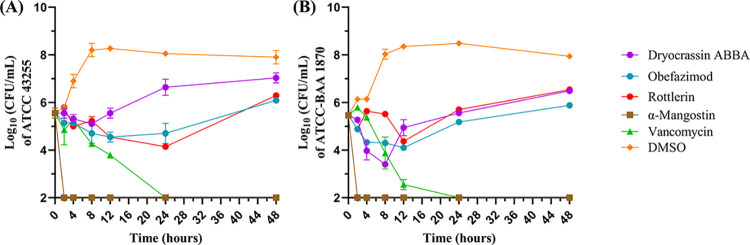
Time-kill assay of most potent hits and control antibiotics against *C*. *difficile*.

To assess whether the antivirals have bactericidal or bacteriostatic killing kinetics activity against *C*. *difficile*, a time-kill assay was performed against *C*. *difficile* ATCC-BAA 1870 and ATCC 43255. As demonstrated in **Fig 2A,** and **2B**, α-mangostin exhibited strong bactericidal activity within 2 hours, significantly better than vancomycin, which demonstrated bactericidal activity at 12 hours for both strains. In contrast, dryocrassin ABBA, rottlerin, and obefazimod displayed bacteriostatic activity with approximately 1–1.5 log_10_ CFU reduction within 12 hours and remained static up to 48 hours (**Fig 2A,** and **2B**).

The growth of *C*. *difficile* ATCC 43255 (A) or ATCC BAA-1870 (B) at was observed as log_10_ CFU/mL over a 24-hour period. The compounds dryocrassin ABBA (purple), obefazimod (blue), rottlerin (red), α-mangostin (brown), vancomycin (green), and DMSO (orange) were tested at 5x their MIC.

## Discussion

*C*. *difficile* is the leading cause of antibiotic-associated diarrhea in the United States, accounting for nearly half a million infections annually [7, 51]. This is in part due to currently available treatment options, vancomycin and fidaxomicin, being associated with significant rates of treatment failure (20–35%) for the initial antibiotic treatment with 40–60% of those cases experiencing recurrence again [13, 52–54]. Moreover, both of the approved drugs continue to pose the risk of *C*. *difficile* acquiring antibiotic resistance, which emphasizes the urgent need for a unique, reliable, and efficient medication or pharmacological scaffold to treat CDI [55]. Many new antivirals have poor oral bioavailability, which is a considerable challenge for these compounds to be approved as drugs or antivirals [56, 57]. Therefore, finding antivirals with potent anti-*C*. *difficile* activity is a fruitful strategy because these drugs could remain longer in the gut where *C*. *difficile* grows and colonizes due to their limited absorption.

In this study, a whole cell-based screening of 618 of antiviral compounds was conducted against *C*. *difficile*. In the initial screening, 24 compounds were found to have anti-*C*. *difficile* activity. After excluding anticancer compounds and previously reported hits, we pursued 9 antivirals for further study against *C*. *difficile*. These results were confirmed and refined, ultimately narrowing down to 4 agents (rottlerin, α-mangostin, dryocrassin ABBA, and obefazimod) that displayed the most potent activity against *C*. *difficile* ATCC BAA-1870 with MIC values ≤4 μM. Due to their unique nature and lack of prior research regarding their use as antimicrobials, these 4 agents were chosen for further investigation. To further confirm their activity, these hits were purchased commercially and have been tested against a panel of 15 clinical isolates of *C*. *difficile* and their MIC_50_ and MIC_90_ were determined.

Rottlerin, also known as mallotoxin, is a natural product isolated from *Mallotus phillippinesis* and is a known protein kinase Cδ (PKCδ)-selective inhibitor and mitochondrial uncoupler that is typically utilized as the base for substrate phosphorylation studies [58, 59]. However, there has been some debate on whether it is selective for protein kinase C [59]. Rottlerin has displayed activity targeting quorum sensing and biofilm activity in *Pseudomonas aeruginosa* [60]. It has also shown activity against *mycobacterium spp*., including *M*. *tuberculosis* and *M*. *smegmatis* (IC_50_ range from 9 to 74 μM), potentially interfering with shikimate kinase, a promising target for antimicrobials, which is essential for many bacteria [61]. It also has been reported to target *Chlamydia* species with MIC values of 1 μM [62]. Recently, rottlerin was found to have some fungicidal activity in a study where MIC and MFC values against *Candida* species were as low as 7.81 μg/mL [63]. Here, we report that rottlerin exhibited strong antibacterial activity against *C*. *difficile* (MIC_50_ = 0.25 μg/mL and MIC_90_ = 0.5 μg/mL), which was comparable to the control antibiotic, vancomycin.

α-mangostin is a xanthone derivative extracted from the edible fruit of *Garcinia mangostana*, commonly known as mangosteen [64]. Interestingly, α-mangostin has faced issues as a treatment due to its lack of absorption and oral bioavailability, leading researchers to modify α-mangostin to improve its pharmacokinetics [65]. Interestingly, α-mangostin has been found in high concentrations within the small intestine, which makes it an intriguing molecule to further pursue for CDI treatment. Both primary treatments for moderate to severe CDI, vancomycin and fidaxomicin, have minimal systemic absorption when taken orally [66, 67]. α-mangostin showed potent anti-*C*. *difficile* activity (MIC_50_ = 1μg/mL and MIC_90_ = 2μg/mL), equivalent to that of the drug of choice, vancomycin, with little to no activity against the microbiota. In addition, α-mangostin has shown potent and rapid bactericidal activity both in this study and against methicillin-resistant *S*. *aureus* (MRSA) [68–70].

Dryocrassin ABBA is a phloroglucinol derivative that is isolated from *Dryopteris crassirhizoma* [71] and has previously been reported to have antimicrobial activity against both H5N1 avian influenza virus and *S*. *aureus* virulence factors [72, 73]. Particularly, it has potent activity against virulence factors of MRSA such as Von Willebrand factor-binding proteins (vWbp) and Sortase A [73, 74]. Otherwise, it does not have activity against *S*. *aureus* itself (MIC >1024 μg/mL) [73]. Herein, dryocrassin ABBA showed activity similar to that of vancomycin against *C*. *difficile*, with an MIC_50_ and MIC_90_ of 1 μg/mL and 4 μg/mL.

Obefazimod, also known as ABX464, is a novel and first-in-class compound developed by Abivax and has shown antiviral, antirheumatic, and anti-colitis properties [49, 75, 76]. It is currently in phase 2 trials for its antiviral and anti-rheumatic properties, while in phase 3 trials for the anti-colitis effect [49, 50, 77, 78]. There have been no previous reports of antibacterial activity published so far, making this compound of great interest as a potential novel class of antimicrobials on top of its other therapeutic effects. This compound had an MIC_50_ of 4 μg/mL and an MIC_90_ of 8 μg/mL, and displayed bacteriostatic activity against *C*. *difficile*. Its activity against human microbiota was lower across the board when compared to control antibiotics apart from *Bacteroides* strains.

*C*. *difficile* is an opportunistic pathogen that is intimately tied with gut dysbiosis. Administration of broad-spectrum antibiotics can disrupt commensal gut microflora, which are considered the first line of defense against *C*. *difficile* colonization [79]. Due to this, selectivity against *C*. *difficile* could be beneficial in preventing recurrence and reducing the severity of CDI [80]. Therefore, it is necessary to take into consideration whether these compounds have an impact on beneficial bacteria typically found in the human gut microbiota. Overall, the selected compounds had very little effect on gut microbiota and, in some cases, had MICs higher than those of drug of choice.

Next, we aimed to monitor the killing kinetics of the selected antiviral compounds to determine whether they had bactericidal or bacteriostatic activity. We found that 3 compounds, rottlerin, dryocrassin ABBA, and obefazimod had bacteriostatic activity. However, α-mangostin displayed rapid bactericidal activity far more potent than both fidaxomicin and vancomycin, achieving 100% inhibition within the first 2 hours. Remarkably, α-mangostin’s potent activity against *C*. *difficile* (MIC_50_ = 1μg/mL and MIC_90_ = 2μg/mL) did not translate to other bacteria with little to no activity against microbiota and a far more rapid mechanism of killing highlighted the potential of this compound for CDI treatment.

To conclude, the goal of this work was to identify the antivirals with notable anti-*C*. *difficile* activity, such as obefazimod, rottlerin, α-mangostin, and dryocrassin ABBA, that could be thus classified as anti-*C*. *difficile* drugs. Most notably, α-mangostin’s killing kinetics were superior to those of the currently approved drugs. Additionally, nearly all drugs have shown little to no efficacy against the commensal microbiota, which is particularly favorable in preventing recurrent CDI. Therefore, these antivirals could function as lead structures for further development of anti-*C*. *difficile* drugs.

## Supporting information

S1 File (DOCX)

S1 Fig (XLSX)

S2 Fig (XLSX)

## References

[1] Prevention CfDCa. Antibiotic resistance threats in the Unites States, 2019. In: Centers for Disease Control and Prevention NCfEZaID, Division of Healthcare Quality Promotion, Antibiotic Resistance Coordination and Strategy Unit, editor. 2019.

[2] Prevention CfDCa. Healthcare-Associated Infections Community Interface Surveillance Report, *Clostridioides difficile* infection (CDI), 2022. In: Program EI, editor.: CDC; 2024.

[3] Feuerstadt P, Nelson WW, Drozd EM, Dreyfus J, Dahdal DN, Wong AC, et al. Mortality, Health Care Use, and Costs of *Clostridioides difficile* Infections in Older Adults. Journal of the American Medical Directors Association. 2022;23(10):1721–8.e19. doi: 10.1016/j.jamda.2022.01.075 35288083

[4] Abutaleb NS, Seleem MN. Auranofin, at clinically achievable dose, protects mice and prevents recurrence from *Clostridioides difficile* infection. Scientific Reports. 2020;10(1):7701. doi: 10.1038/s41598-020-64882-9 32382070 PMC7206065

[5] Abutaleb NS, Seleem MN. In vivo efficacy of auranofin in a hamster model of *Clostridioides difficile* infection. Scientific Reports. 2021;11(1):7093. doi: 10.1038/s41598-021-86595-3 33782498 PMC8007812

[6] Laszkowska M, Kim J, Faye AS, Joelson AM, Ingram M, Truong H, et al. Prevalence of *Clostridioides difficile* and Other Gastrointestinal Pathogens in Patients with COVID-19. Digestive Diseases and Sciences. 2021;66(12):4398–405. doi: 10.1007/s10620-020-06760-y 33479861 PMC7819769

[7] Leffler DA, Lamont JT. *Clostridium difficile* Infection. New England Journal of Medicine. 2015;372(16):1539–48. doi: 10.1056/NEJMra1403772 WOS:000352856500009. 25875259

[8] Mullish BH, Williams HRT. *Clostridium difficile* infection and antibiotic-associated diarrhoea. Clinical Medicine. 2018;18(3):237–41. doi: 10.7861/clinmedicine.18-3-237 WOS:000434906300009. 29858434 PMC6334067

[9] Pal R, Athamneh AIM, Deshpande R, Ramirez JAR, Adu KT, Muthuirulan P, et al. Probiotics: insights and new opportunities for *Clostridioides difficile* intervention. Critical Reviews in Microbiology. 2023;49(3):414–34. doi: 10.1080/1040841X.2022.2072705 35574602 PMC9743071

[10] McDonald LC, Gerding DN, Johnson S, Bakken JS, Carroll KC, Coffin SE, et al. Clinical Practice Guidelines for *Clostridium difficile* Infection in Adults and Children: 2017 Update by the Infectious Diseases Society of America (IDSA) and Society for Healthcare Epidemiology of America (SHEA). Clinical Infectious Diseases. 2018;66(7):e1–e48. doi: 10.1093/cid/cix1085 29462280 PMC6018983

[11] McDonald LC, Gerding DN, Johnson S, Bakken JS, Carroll KC, Coffin SE, et al. Clinical Practice Guidelines for *Clostridium difficile* Infection in Adults and Children: 2017 Update by the Infectious Diseases Society of America (IDSA) and Society for Healthcare Epidemiology of America (SHEA). Clinical Infectious Diseases. 2018;66(7):987–94. doi: 10.1093/cid/ciy149 WOS:000427897000006. 29562266

[12] Tieu JD, Williams RJ, Skrepnek GH, Gentry CA. Clinical outcomes of fidaxomicin vs oral vancomycin in recurrent *Clostridium difficile* infection. Journal of Clinical Pharmacy and Therapeutics. 2019;44(2):220–8. doi: 10.1111/jcpt.12771 WOS:000460316100011. 30350418

[13] Spiceland CM, Khanna S, Pardi DS. Outcomes With Fidaxomicin Therapy in *Clostridium difficile* Infection. Journal of Clinical Gastroenterology. 2018;52(2):151–4. doi: 10.1097/mcg.0000000000000769 WOS:000423453900011. 28009682

[14] De Clercq E, Li GD. Approved Antiviral Drugs over the Past 50 Years. Clinical Microbiology Reviews. 2016;29(3):695–747. doi: 10.1128/cmr.00102-15 WOS:000382397800005. 27281742 PMC4978613

[15] Chen R, Wang TT, Song J, Pu DJ, He D, Li JJ, et al. Antiviral Drug Delivery System for Enhanced Bioactivity, Better Metabolism and Pharmacokinetic Characteristics. International Journal of Nanomedicine. 2021;16:4959–84. doi: 10.2147/IJN.S315705 WOS:000678334800003. 34326637 PMC8315226

[16] Razonable RR, editor Antiviral drugs for viruses other than human immunodeficiency virus. Mayo Clinic Proceedings; 2011: Elsevier. 10.4065/mcp.2011.0309 PMC3184032 21964179

[17] Kociolek LK, Gerding DN. Breakthroughs in the treatment and prevention of *Clostridium difficile* infection. Nature Reviews Gastroenterology & Hepatology. 2016;13(3):150–60. doi: 10.1038/nrgastro.2015.220 26860266

[18] Brouwer DM, Corallo CE, Coutsouvelis J. Systemic Absorption of Low‐Dose Oral Vancomycin. Journal of Pharmacy Practice and Research. 2005;35.

[19] Shue YK, Sears PS, Shangle S, Walsh RB, Lee C, Gorbach SL, et al. Safety, tolerance, and pharmacokinetic studies of OPT-80 in healthy volunteers following single and multiple oral doses. Antimicrob Agents Chemother. 2008;52(4):1391–5. Epub 20080211. doi: 10.1128/AAC.01045-07 ; PubMed Central PMCID: PMC2292557. 18268081 PMC2292557

[20] Rossignol JF. Nitazoxanide: A first-in-class broad-spectrum antiviral agent. Antiviral Research. 2014;110:94–103. doi: 10.1016/j.antiviral.2014.07.014 WOS:000343352600011. 25108173 PMC7113776

[21] Musher DM, Logan N, Bressler AM, Johnson DP, Rossignol JF. Nitazoxanide versus Vancomycin in *Clostridium difficile* Infection: A Randomized, Double-Blind Study. Clinical Infectious Diseases. 2009;48(4):E41–E6. doi: 10.1086/596552 WOS:000262585700027. 19133801

[22] AbdelKhalek A, Mohammad H, Mayhoub AS, Seleem MN. Screening for potent and selective anticlostridial leads among FDA-approved drugs. Journal of Antibiotics. 2020;73(6):392–409. doi: 10.1038/s41429-020-0288-3 WOS:000518095400002. 32132676 PMC7211121

[23] Wang GZ, Li L, Wang XK, Li X, Zhang YW, Yu J, et al. Hypericin enhances β-lactam antibiotics activity by inhibiting sarA expression in methicillin-resistant Staphylococcus aureus. Acta Pharmaceutica Sinica B. 2019;9(6):1174–82. doi: 10.1016/j.apsb.2019.05.002 WOS:000500912700006. 31867163 PMC6900551

[24] Abouelkhair AA, Seleem MN. Exploring novel microbial metaoblites and drugs for inhibiting *Clostridioides difficile*. mSphere. 2024. doi: 10.1128/msphere.00273-24 38940508 PMC11288027

[25] Pal R, Seleem MN. Screening of Natural Products and Approved Oncology Drug Libraries for Activity against *Clostridioides difficile*. Scientific Reports. 2020;10(1). doi: 10.1038/s41598-020-63029-0 WOS:000563471200007. 32249833 PMC7136261

[26] Pal R, Seleem MN. Discovery of a novel natural product inhibitor of *Clostridioides difficile* with potent activity in vitro and in vivo. Plos One. 2022;17(8). doi: 10.1371/journal.pone.0267859 WOS:000933388000005. 35939437 PMC9359557

[27] AbdelKhalek A, Abutaleb NS, Mohammad H, Seleem MN. Antibacterial and antivirulence activities of auranofin against *Clostridium difficile*. International Journal of Antimicrobial Agents. 2019;53(1):54–62. doi: 10.1016/j.ijantimicag.2018.09.018 WOS:000455090800009. 30273668 PMC6475173

[28] Pal R, Dai M, Seleem MN. High-throughput screening identifies a novel natural product-inspired scaffold capable of inhibiting *Clostridioides difficile* in vitro. Scientific Reports. 2021;11(1):10913. doi: 10.1038/s41598-021-90314-3 34035338 PMC8149678

[29] AbdelKhalek A, Seleem MN. Repurposing the Veterinary Antiprotozoal Drug Ronidazole for the Treatment of *Clostridioides difficile* Infection. International Journal of Antimicrobial Agents. 2020;56(6). doi: 10.1016/j.ijantimicag.2020.106188 WOS:000596387600023. 33045352 PMC7704610

[30] Naclerio GA, Abutaleb NS, Li DY, Seleem MN, Sintim HO. Ultrapotent Inhibitor of *Clostridioides difficile* Growth, Which Suppresses Recurrence In Vivo. Journal of Medicinal Chemistry. 2020;63(20):11934–44. doi: 10.1021/acs.jmedchem.0c01198 WOS:000585952100034. 32960605 PMC9064041

[31] Shao XW, AbdelKhalek A, Abutaleb NS, Velagapudi UK, Yoganathan S, Seleem MN, et al. Chemical Space Exploration around Thieno 3,2-d pyrimidin-4(3H)-one Scaffold Led to a Novel Class of Highly Active *Clostridium difficile* Inhibitors. Journal of Medicinal Chemistry. 2019;62(21):9772–91. doi: 10.1021/acs.jmedchem.9b01198 WOS:000497260700026. 31584822

[32] Pal R, Seleem MN. Antisense inhibition of RNA polymerase α subunit of *Clostridioides difficile*. Microbiology Spectrum. 2023;11(5). doi: 10.1128/spectrum.01755-23 WOS:001107303900129. 37772833 PMC10581251

[33] Mody D, Athamneh AIM, Seleem MN. Curcumin: A natural derivative with antibacterial activity against *Clostridium difficile*. Journal of Global Antimicrobial Resistance. 2020;21:154–61. doi: 10.1016/j.jgar.2019.10.005 WOS:000544879300029. 31622683 PMC7153983

[34] Abutaleb NS, Seleem MN. Repurposing the Antiamoebic Drug Diiodohydroxyquinoline for Treatment of *Clostridioides difficile* Infections. Antimicrobial Agents and Chemotherapy. 2020;64(6):10.1128/aac.02115-19. doi: 10.1128/aac.02115-19 32253206 PMC7269495

[35] Carvalho GM, Silva BA, Xavier RGC, Zanon IP, Vilela EG, Nicolino RR, et al. Evaluation of disk diffusion method for testing the rifampicin, erythromycin, and tetracycline susceptibility of Clostridioides (prev. Clostridium) difficile. Anaerobe. 2023;80:102720. doi: 10.1016/j.anaerobe.2023.102720 36934966

[36] McVay Catherine S, Rolfe Rial D. In Vitro and In Vivo Activities of Nitazoxanide against *Clostridium difficile*. Antimicrobial Agents and Chemotherapy. 2000;44(9):2254–8. doi: 10.1128/aac.44.9.2254–2258.2000 10952564 PMC90054

[37] Pankuch Glenn A, Appelbaum Peter C. Activities of Tizoxanide and Nitazoxanide Compared to Those of Five Other Thiazolides and Three Other Agents against Anaerobic Species. Antimicrobial Agents and Chemotherapy. 2006;50(3):1112–7. doi: 10.1128/AAC.50.3.1112-1117.2006 16495282 PMC1426457

[38] Cermak P, Olsovska J, Mikyska A, Dusek M, Kadleckova Z, Vanicek J, et al. Strong antimicrobial activity of xanthohumol and other derivatives from hops (Humulus lupulus L.) on gut anaerobic bacteria. Apmis. 2017;125(11):1033–8. doi: 10.1111/apm.12747 28960474

[39] Sleha R, Radochova V, Mikyska A, Houska M, Bolehovska R, Janovska S, et al. Strong antimicrobial effects of xanthohumol and beta-acids from hops against *clostridioides difficile* infection in vivo. Antibiotics. 2021;10(4):392. 33917416 10.3390/antibiotics10040392 PMC8067520

[40] Wu X, Alam M, Feng L, Tsutsumi LS, Sun D, Hurdle JG. Prospects for flavonoid and related phytochemicals as nature‐inspired treatments for *Clostridium difficile* infection. Journal of applied microbiology. 2014;116(1):23–31. 24479135 10.1111/jam.12344 PMC3910234

[41] Li Y, She P, Xu L, Liu Y, Liu S, Li Z, et al. Anti-hepatitis C virus drug simeprevir: a promising antimicrobial agent against MRSA. Applied Microbiology and Biotechnology. 2022;106(7):2689–702. doi: 10.1007/s00253-022-11878-2 35338386

[42] Lin G, Voss K, Davidson TJ. Acute inhalation toxicity of cetylpyridinium chloride. Food and chemical toxicology. 1991;29(12):851–4. doi: 10.1016/0278-6915(91)90113-l 1765331

[43] Wang F, Gan T, Chou TF. Allosteric p97 inhibitors to overcome ATP‐competitive inhibitors resistance in anticancer therapy. The FASEB Journal. 2018;32:807.6-.6.29018142

[44] Wang Q, Shinkre BA, Lee J-g, Weniger MA, Liu Y, Chen W, et al. The ERAD Inhibitor Eeyarestatin I Is a Bifunctional Compound with a Membrane-Binding Domain and a p97/VCP Inhibitory Group. PLOS ONE. 2010;5(11):e15479. doi: 10.1371/journal.pone.0015479 21124757 PMC2993181

[45] Wang Q, Mora-Jensen H, Weniger MA, Perez-Galan P, Wolford C, Hai T, et al. ERAD inhibitors integrate ER stress with an epigenetic mechanism to activate BH3-only protein NOXA in cancer cells. Proceedings of the National Academy of Sciences. 2009;106(7):2200–5. doi: 10.1073/pnas.0807611106 19164757 PMC2629785

[46] Zhang G, Li S, Cheng K-W, Chou T-F. AAA ATPases as therapeutic targets: Structure, functions, and small-molecule inhibitors. European Journal of Medicinal Chemistry. 2021;219:113446. doi: 10.1016/j.ejmech.2021.113446 33873056 PMC8165034

[47] Zhang G, Li S, Wang F, Jones AC, Goldberg AFG, Lin B, et al. A covalent p97/VCP ATPase inhibitor can overcome resistance to CB-5083 and NMS-873 in colorectal cancer cells. European Journal of Medicinal Chemistry. 2021;213:113148. doi: 10.1016/j.ejmech.2020.113148 33476933 PMC7954469

[48] Chou T-F, Li K, Frankowski KJ, Schoenen FJ, Deshaies RJ. Structure–Activity Relationship Study Reveals ML240 and ML241 as Potent and Selective Inhibitors of p97 ATPase. ChemMedChem. 2013;8(2):297–312. doi: 10.1002/cmdc.201200520 23316025 PMC3662613

[49] Bernal S, Puertas MC, Morón-López S, Cranston RD, Urrea V, Dalmau J, et al. Impact of Obefazimod on Viral Persistence, Inflammation, and Immune Activation in People With Human Immunodeficiency Virus on Suppressive Antiretroviral Therapy. Journal of Infectious Diseases. 2023;228(9):1280–91. doi: 10.1093/infdis/jiad251 WOS:001030706700001. 37395474 PMC10629703

[50] Santo J, Flatres A, Gineste P, Scherrer D, Nitcheu J, Sloan S, et al. Correlation of miR-124 Upregulation and PK Parameters in Blood of Patients With Moderate-to-Severe Ulcerative Colitis Receiving Obefazimod for 16 Weeks. American Journal of Gastroenterology. 2023;118(10):S836–S. WOS:001091849302213.

[51] Eeuwijk J, Ferreira G, Yarzabal JP, Robert-Du Ry van Beest Holle M. A Systematic Literature Review on Risk Factors for and Timing of *Clostridioides difficile* Infection in the United States. Infect Dis Ther. 2024;13(2):273–98. Epub 20240213. doi: 10.1007/s40121-024-00919-0 ; PubMed Central PMCID: PMC10904710. 38349594 PMC10904710

[52] Cornely OA, Miller MA, Louie TJ, Crook DW, Gorbach SL. Treatment of first recurrence of *Clostridium difficile* infection: fidaxomicin versus vancomycin. Clin Infect Dis. 2012;55 Suppl 2(Suppl 2):S154–61. doi: 10.1093/cid/cis462 ; PubMed Central PMCID: PMC3388030. 22752865 PMC3388030

[53] McFarland LV, Elmer GW, Surawicz CM. Breaking the cycle: treatment strategies for 163 cases of recurrent *Clostridium difficile* disease. Am J Gastroenterol. 2002;97(7):1769–75. doi: 10.1111/j.1572-0241.2002.05839.x . 12135033

[54] Hopkins RJ, Wilson RB. Treatment of recurrent *Clostridium difficile* colitis: a narrative review. Gastroenterol Rep (Oxf). 2018;6(1):21–8. Epub 20171218. doi: 10.1093/gastro/gox041 ; PubMed Central PMCID: PMC5806400. 29479439 PMC5806400

[55] Spigaglia P, Mastrantonio P, Barbanti F. Antibiotic Resistances of *Clostridium difficile*. In: Mastrantonio P, Rupnik M, editors. Updates on *Clostridium Difficile* in Europe: Advances in Microbiology, Infectious Diseases and Public Health, Vol 8. Advances in Experimental Medicine and Biology. 10502018. p. 137–59.

[56] Righi E, Lambertenghi L, Gorska A, Sciammarella C, Ivaldi F, Mirandola M, et al. Impact of COVID-19 and Antibiotic Treatments on Gut Microbiome: A Role for Enterococcus spp. Biomedicines. 2022;10(11). doi: 10.3390/biomedicines10112786 WOS:000880931500001. 36359311 PMC9687172

[57] Chedid M, Waked R, Haddad E, Chetata N, Saliba G, Choucair J. Antibiotics in treatment of COVID-19 complications: a review of frequency, indications, and efficacy. Journal of Infection and Public Health. 2021;14(5):570–6. doi: 10.1016/j.jiph.2021.02.001 WOS:000646776000003. 33848886 PMC7870433

[58] Gschwendt HJM M., Kielbassa K., Zang R., Kittstein W., Rincke G., Marks F. Rottlerin, a Novel Protein Kinase Inhibitor. Biochemical and Biophysical Research Communications. 1994;199(1):93–8. Epub 25 May 2002. doi: 10.1006/bbrc.1994.1199 8123051

[59] Soltoff SP. Rottlerin:: an inappropriate and ineffective inhibitor of PKCδ. Trends in Pharmacological Sciences. 2007;28(9):453–8. doi: 10.1016/j.tips.2007.07.003 WOS:000249932800003. 17692392

[60] Suresh D, Sabir S, Yu TT, Wenholz D, Das T, Black DS, et al. Natural Product Rottlerin Derivatives Targeting Quorum Sensing. Molecules. 2021;26(12). doi: 10.3390/molecules26123745 WOS:000665978900001. 34205355 PMC8235494

[61] Pandey S, Chatterjee A, Jaiswal S, Kumar S, Ramachandran R, Srivastava KK. Protein kinase C-δ inhibitor, Rottlerin inhibits growth and survival of mycobacteria exclusively through Shikimate kinase. Biochemical and Biophysical Research Communications. 2016;478(2):721–6. doi: 10.1016/j.bbrc.2016.08.014 WOS:000383528600034. 27498028

[62] Shivshankar P, Lei L, Wang J, Zhong G. Rottlerin Inhibits Chlamydial Intracellular Growth and Blocks Chlamydial Acquisition of Sphingolipids from Host Cells. Applied and Environmental Microbiology. 2008;74(4):1243–9. doi: 10.1128/AEM.02151-07 18083882 PMC2258579

[63] Silva NBS, Menezes RP, Gonçalves DS, Santiago MB, Conejo NC, Souza SL, et al. Exploring the antifungal, antibiofilm and antienzymatic potential of Rottlerin in an in vitro and in vivo approach. Scientific Reports. 2024;14(1):11132. doi: 10.1038/s41598-024-61179-z 38750088 PMC11096346

[64] Ghasemzadeh A, Jaafar HZE, Baghdadi A, Tayebi-Meigooni A. Alpha-Mangostin-Rich Extracts from Mangosteen Pericarp: Optimization of Green Extraction Protocol and Evaluation of Biological Activity. Molecules. 2018;23(8). doi: 10.3390/molecules23081852 WOS:000445295500017. 30044450 PMC6222712

[65] Ibrahim MY, Hashim NM, Mariod AA, Mohan S, Abdulla MA, Abdelwahab SI, et al. α-Mangostin from Garcinia mangostana Linn: An updated review of its pharmacological properties. Arabian Journal of Chemistry. 2016;9(3):317–29. doi: 10.1016/j.arabjc.2014.02.011 WOS:000375599500001.

[66] Johnson S, Louie TJ, Gerding DN, Cornely OA, Chasan-Taber S, Fitts D, et al. Vancomycin, Metronidazole, or Tolevamer for *Clostridium difficile* Infection: Results From Two Multinational, Randomized, Controlled Trials. Clinical Infectious Diseases. 2014;59(3):345–54. doi: 10.1093/cid/ciu313 WOS:000342919700004. 24799326

[67] Cherian PT, Wu XQ, Yang L, Scarborough JS, Singh AP, Alam ZA, et al. Gastrointestinal localization of metronidazole by a lactobacilli-inspired tetramic acid motif improves treatment outcomes in the hamster model of *Clostridium difficile* infection. Journal of Antimicrobial Chemotherapy. 2015;70(11):3061–9. doi: 10.1093/jac/dkv231 WOS:000368245500018. 26286574 PMC4677261

[68] Sivaranjani M, Prakash M, Gowrishankar S, Rathna J, Pandian SK, Ravi AV. In vitro activity of alpha-mangostin in killing and eradicating Staphylococcus epidermidis RP62A biofilms. Applied Microbiology and Biotechnology. 2017;101(8):3349–59. doi: 10.1007/s00253-017-8231-7 WOS:000399167800028. 28343241

[69] Koh JJ, Qiu SX, Zou HX, Lakshminarayanan R, Li JG, Zhou XJ, et al. Rapid bactericidal action of alpha-mangostin against MRSA as an outcome of membrane targeting. Biochimica Et Biophysica Acta-Biomembranes. 2013;1828(2):834–44. doi: 10.1016/j.bbamem.2012.09.004 WOS:000315004600073. 22982495

[70] Deng XB, Xu HB, Li DY, Chen JL, Yu ZJ, Deng QW, et al. Mechanisms of Rapid Bactericidal and Anti-Biofilm Alpha-Mangostin In Vitro Activity against Staphylococcus aureus. Polish Journal of Microbiology. 2023;72(2):199–208. doi: 10.33073/pjm-2023-021 WOS:001009828200010. 37314356 PMC10266293

[71] Hou B, Liu Z, Yang XB, Zhu WF, Li JY, Yang L, et al. Total synthesis of dryocrassin ABBA and its analogues with potential inhibitory activity against drug-resistant neuraminidases. Bioorganic & Medicinal Chemistry. 2019;27(17):3846–52. doi: 10.1016/j.bmc.2019.07.013 WOS:000477773100010. 31324565

[72] Ou CB, Zhang Q, Wu GJ, Shi NN, He C. Dryocrassin ABBA, a novel active substance for use against amantadine-resistant H5N1 avian influenza virus. Frontiers in Microbiology. 2015;6. doi: 10.3389/fmicb.2015.00592 WOS:000356386600002. 26136733 PMC4468825

[73] Li BB, Jin YL, Xiang H, Mu D, Yang PP, Li XM, et al. An Inhibitory Effect of Dryocrassin ABBA on Staphylococcus aureus vWbp That Protects Mice From Pneumonia. Frontiers in Microbiology. 2019;10. doi: 10.3389/fmicb.2019.00007 WOS:000456501000001. 30728809 PMC6351477

[74] Zhang B, Wang XY, Wang L, Chen SY, Shi DX, Wang HS. Molecular Mechanism of the Flavonoid Natural Product Dryocrassin ABBA against Staphylococcus aureus Sortase A. Molecules. 2016;21(11). doi: 10.3390/molecules21111428 WOS:000389918200012. 27792196 PMC6273746

[75] Thorley J. Obefazimod in rheumatoid arthritis. Lancet Rheumatology. 2022;4(7):E466-E. WOS:000836456100018.

[76] Apolit C, Campos N, Vautrin A, Begon-Pescia C, Lapasset L, Scherrer D, et al. ABX464 (Obefazimod) Upregulates miR-124 to Reduce ProinflammatoryMarkers in Inflammatory Bowel Diseases. Clinical and Translational Gastroenterology. 2023;14(4). doi: 10.14309/ctg.0000000000000560 WOS:001135627100003. 36573890 PMC10132720

[77] Santo J, Gineste P, Scherrer D, Nitcheu J, Ehrlich H, Sands BE, et al. Obefazimod upregulates miR-124 and downregulates the expression of some cytokines in blood and rectal biopsies of patients with moderate-to-severe ulcerative colitis. Journal of Crohns & Colitis. 2023;17:I169–I70. WOS:000960367600132.

[78] Daien C, Krogulec M, Gineste P, Steens JM, du Roure LD, Biguenet S, et al. Safety and efficacy of the miR-124 upregulator ABX464 (obefazimod, 50 and 100 mg per day) in patients with active rheumatoid arthritis and inadequate response to methotrexate and/or anti-TNFα therapy: a placebo-controlled phase II study. Annals of the Rheumatic Diseases. 2022;81(8):1076–84. doi: 10.1136/annrheumdis-2022-222228 WOS:000804668300001. 35641124 PMC9279835

[79] Cao XY, Landick R, Campbell EA. A roadmap for designing narrow-spectrum antibiotics targeting bacterial pathogens. Microbial Cell. 2022;9(7):136–8. doi: 10.15698/mic2022.07.780 WOS:000931720800001. 35855392 PMC9251626

[80] Terrier MCZ, Simonet ML, Bichard P, Frossard JL. Recurrent *Clostridium difficile* infections: The importance of the intestinal microbiota. World Journal of Gastroenterology. 2014;20(23):7416–23. doi: 10.3748/wjg.v20.i23.7416 WOS:000338519800031. 24966611 PMC4064086

