# Supplementary material for: Screening novel antiviral compounds to treat Clostridioides difficile infections
Source: PLoS One. 2024 Dec 13;19(12):e0309624. doi: 10.1371/journal.pone.0309624 (PMC11642915; doi:10.1371/journal.pone.0309624)
Supplement: S1 File — (DOCX) [file pone.0309624.s001.docx]

**S1 Table. Minimum inhibitory concentration (MICs [μM]) of excluded hits against *C. difficile* ATCC BAA-1870 lacking potential.**

| No. | Previously Reported | Structure* | Class | MICs (µM) |
| --- | --- | --- | --- | --- |
| 1 | Rifampicin | 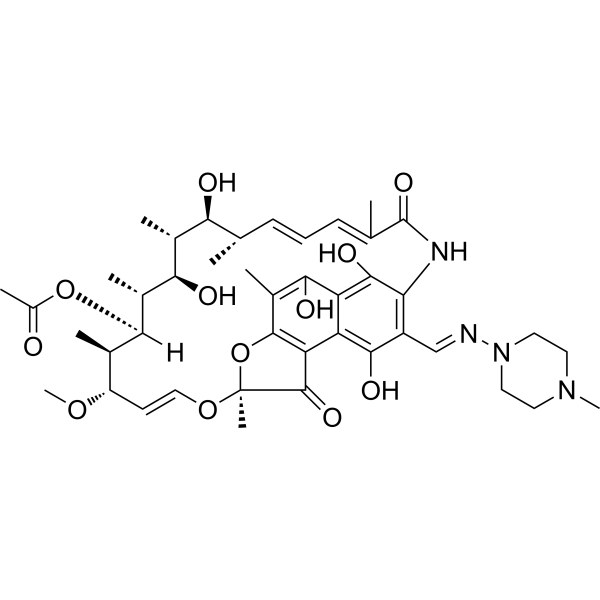 | Antibiotic | <0.125 |
| 2 | Nitazoxanide | 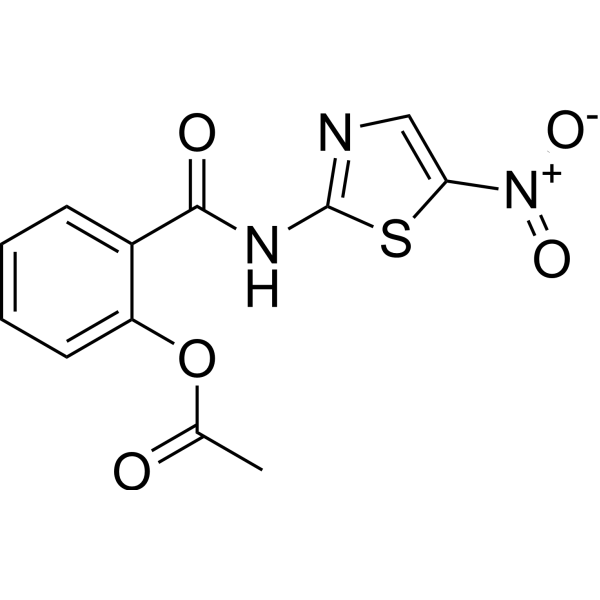 | Antiparasitic & viral | 0.25 |
| 3 | Tizoxanide | 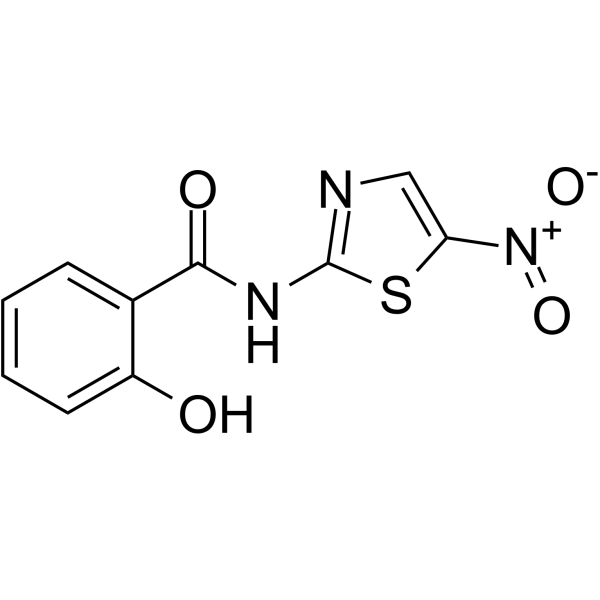 | Antiparasitic | 0.5 |
| 4 | Oxytetracycline (hydrochloride) | 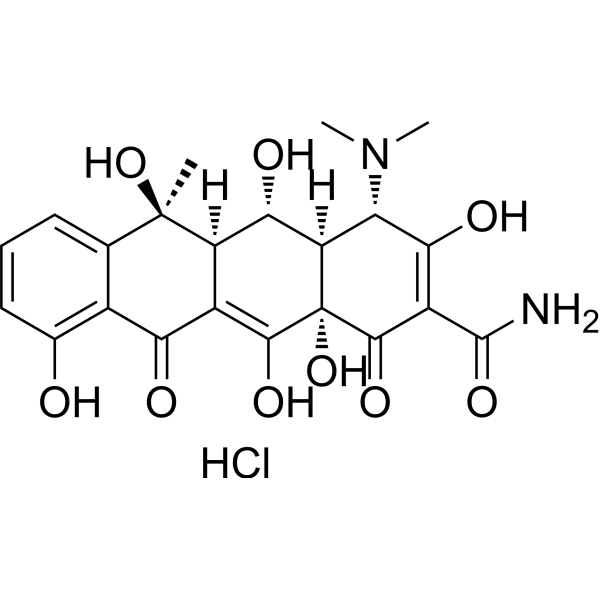 | Antibiotic | 0.5 |
| 5 | Oxytetracycline | 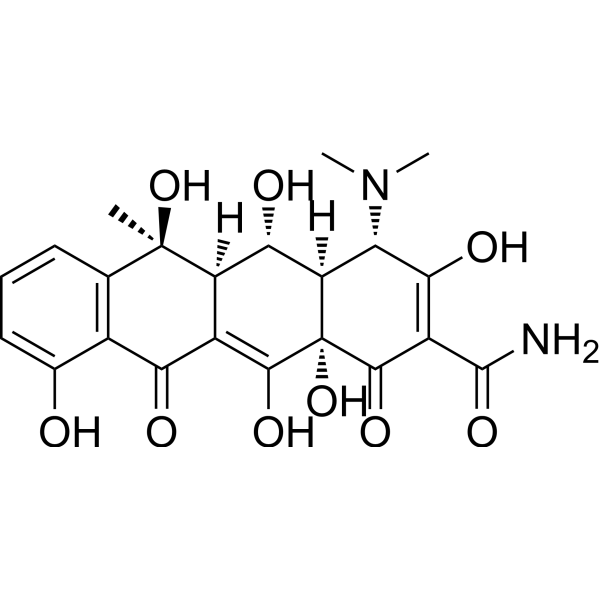 | Antibiotic | 1 |
| 6 | Auranofin | 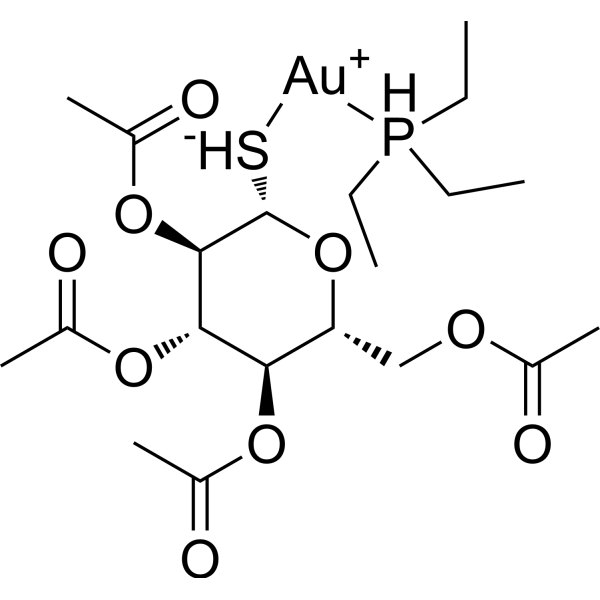 | Antirheumatic | 2 |
| 7 | Xanthohumol | 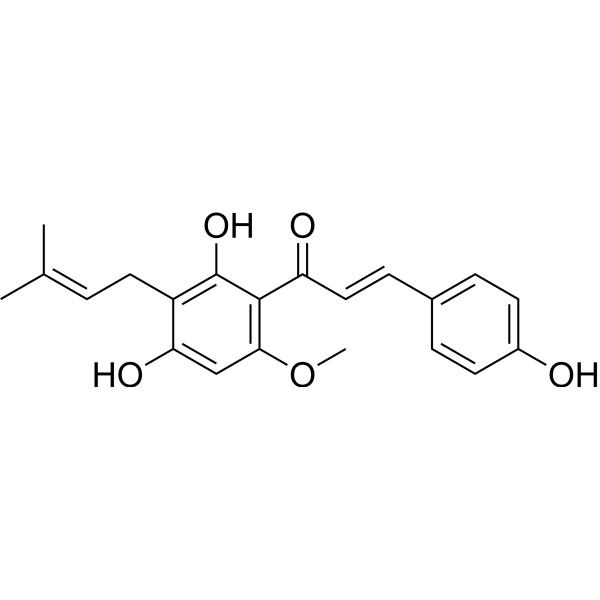 | Natural Product isolated from *Humulus lupulus* | 8 |
| 8 | Kaempferol | 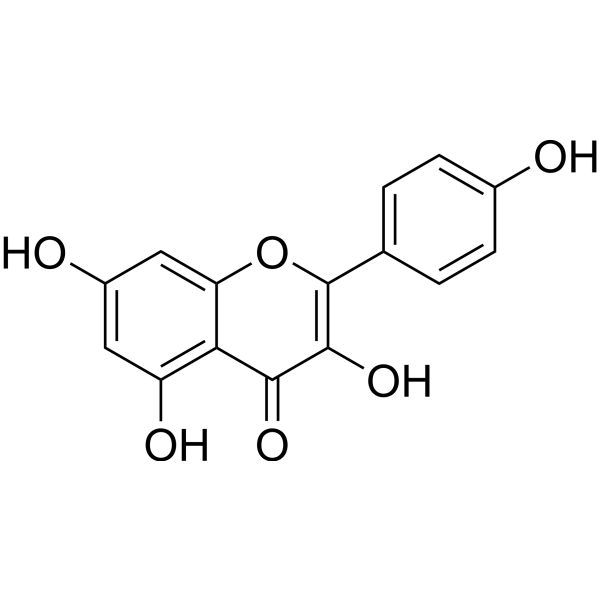 | Natural flavanol | 8 |
| 9 | Simeprevir | 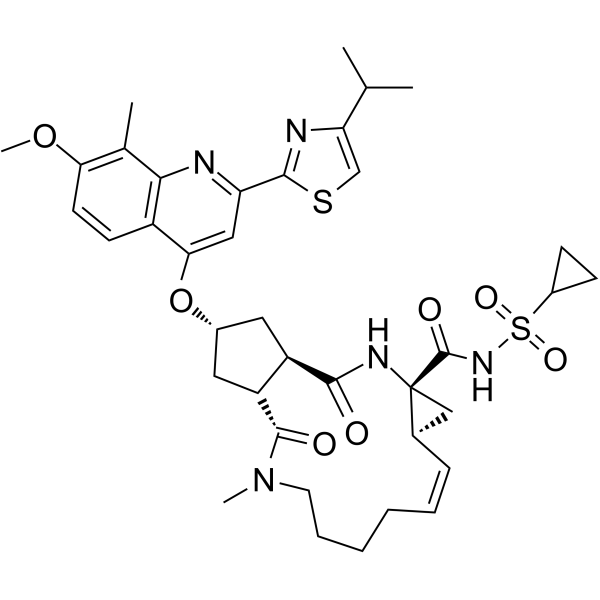 | Antiviral | 8 |
| No. | **Antiseptic and Anticancer Compounds** | **Structure** | **Class** | **MICs (µM)** |
| 10 | NH125 | 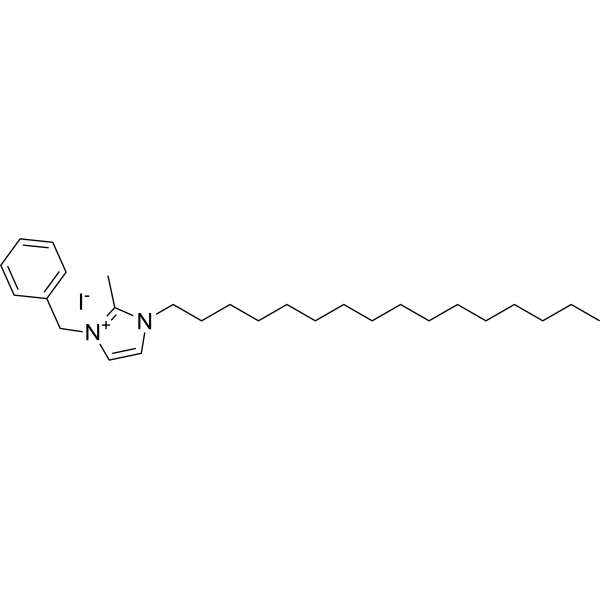 | Antimicrobial Peptide-inspired molecule | 2 |
| 11 | eeyarestatin I | 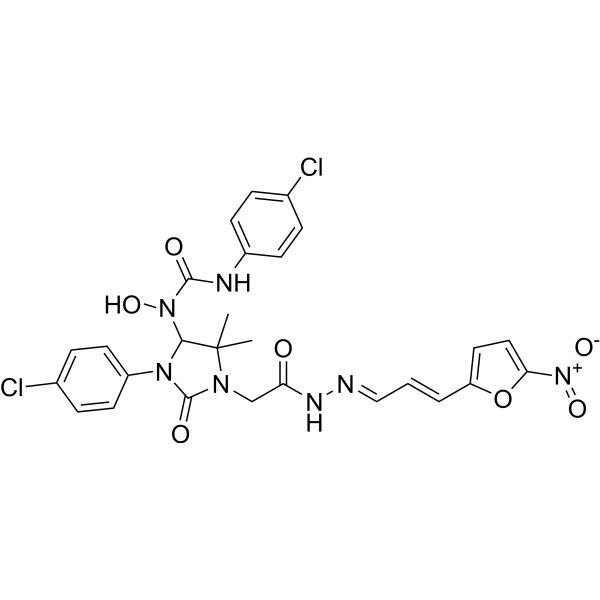 | Anticancer and Endoplasmic Reticulum Associated Protein Degradation (ERAD) Inhibitor | 2 |
| 12 | Cetylpyridinium (Chloride) | 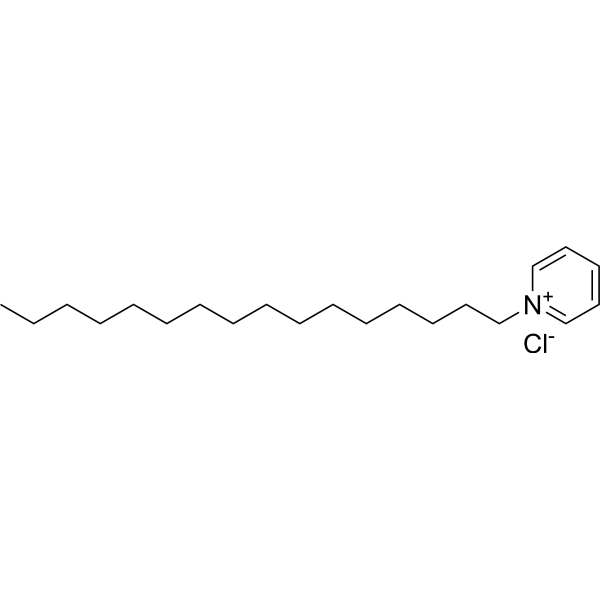 | Antiseptic | 4 |
| 13 | MSC1094308 | 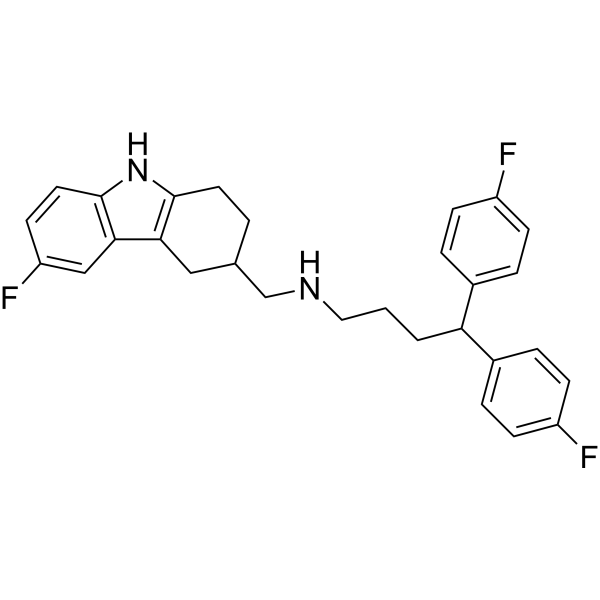 | Anticancer | 8 |
| 14 | ML240 | 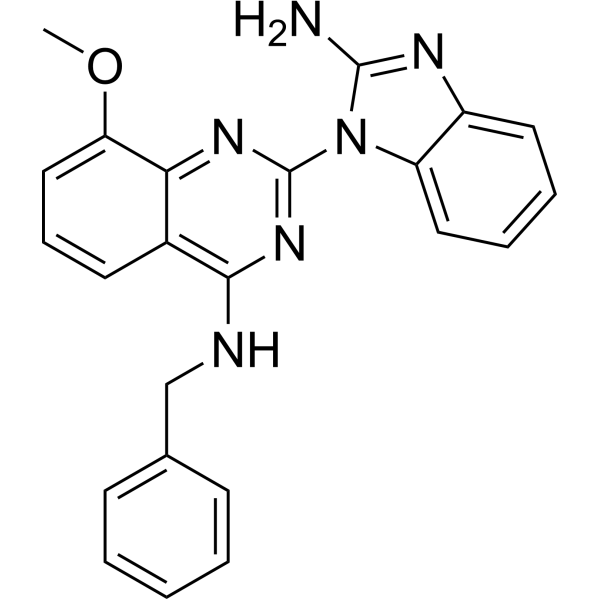 | Anticancer | 8 |
| 15 | TAK-779 | 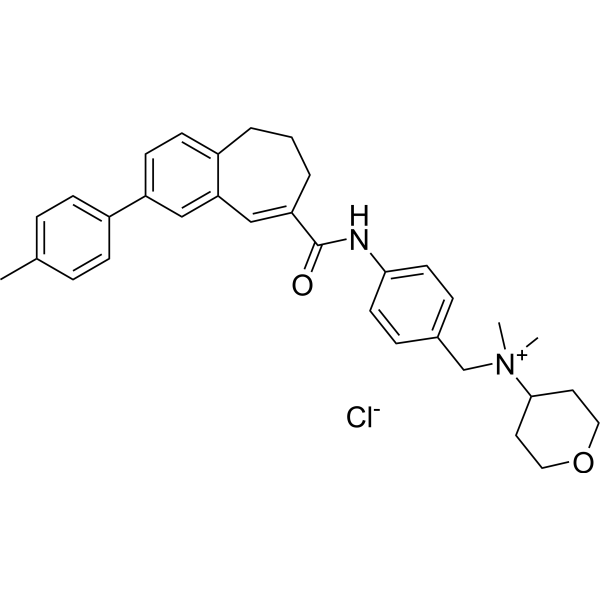 | Anticancer | 16 |

**S2 Table. Full description, sources, and characteristics of *C. difficile* strains.**

| No. | *C. difficile Strain ID* | Alternate Designation | Source | Characteristics |
| --- | --- | --- | --- | --- |
| 1 | **ATCC-BAA 1870** | *Clostridium difficile* Isolate 4118 | Sourced from ATCC. Clinical Isolate (Ribotype – 027) | Positive for *tcdA, tcdB,* and *cdtB* |
| 2 | **ATCC 43255** | *Clostridium difficile* Isolate VPI 10463 | Sourced from ATCC. Isolated from abdominal wound | Positive for *tcdA, tcdB*. Negative for *cdtB*. |
| 3 | **ATCC 43598** | *Clostridium difficile* Isolate 1470 | Sourced from ATCC. Isolated from Belgium from stool of asymptomatic neonate. | Positive for *tcdA, tcdB*. Negative for *cdtB*. |
| 4 | **BAA 1871** | *Clostridium difficile* strain 4111 | Sourced from ATCC. | Positive for *tcdA, tcdB*. Negative for *cdtB*. |
| 5 | **ATCC 630** | *Clostridium difficile* BAA-1382 | Sourced from ATCC. | Positive for *tcdA, tcdB*. Negative for *cdtB*. |
| 6 | **NR-49288** | *Clostridium difficile* Isolate 20110870 | Sourced from BEI Resources.  Isolated from of a young adult female with HA CDI. | Positive for *tcdA, tcdB, tcdC* and *cdtB*. |
| 7 | **NR-49308** | *Clostridium difficile* Isolate 20120166 | Sourced from BEI Resources.  Isolated from of an elderly female with CA CDI. | Positive for *tcdA, tcdB, tcdC.* Negative for CDT. |
| 8 | **NR-49319** | *Clostridium difficile* Isolate 20110992 | Sourced from BEI Resources.  Isolated from of an elderly male with CA CDI. | Positive for *tcdA, tcdB, tcdC.* Negative for CDT. |
| 9 | **NR-49313** | *Clostridium difficile* Isolate 20110963 | Sourced from BEI Resources.  Isolated from of an elderly male with HA CDI. | Positive for *tcdA, tcdB, tcdC.* Negative for CDT. |
| 10 | **NR-49302** | *Clostridium difficile* Isolate 20111075 | Sourced from BEI Resources.  Isolated from of an elderly male with HA CDI. | Positive for *tcdA, tcdB, tcdC.* Negative for CDT. |
| 11 | **CDI-12** | *Clostridium difficile* Isolate 1078 | Sourced from the CDC. Isolated from unknown patient in 2016, USA. | Positive for *tcdA, tcdB,* Negative for CDT. |
| 12 | **CDI-16** | *Clostridium difficile* Isolate 1082 | Sourced from the CDC. Isolated from unknown patient in 2016, USA. | Positive for *tcdA, tcdB,* Negative for CDT. |
| 13 | **CDI-20** | *Clostridium difficile* Isolate 1086 | Sourced from the CDC. Isolated from unknown patient in 2016, USA. | Positive for *tcdA, tcdB,* Negative for CDT. |
| 14 | **CDI-21** | *Clostridium difficile* Isolate 1087 | Sourced from the CDC. Isolated from unknown patient in 2016, USA. | Positive for *tcdA, tcdB,* Negative for CDT. |
| 15 | **CDI-28** | *Clostridium difficile* Isolate 1094 | Sourced from the CDC. Isolated from unknown patient in 2016, USA. | Positive for *tcdA, tcdB,* Positive for CDT. |

**ATCC**: The American Type Culture Collection

**BEI Resources:** The Biodefense and Emerging Infections Resources Repository

**CDC:** The Center for Disease Control and Prevention

**HA:** Hospital acquired

**CA:** Community acquired

**S3 Table. Full description, sources, and characteristics of human microbiota strains.**

| No. | *Bacterial Strain ID* | Alternate Designation | Source |
| --- | --- | --- | --- |
| 1 | ***Levilactobacillus brevis* ATCC-14869** | *Levilactobacillus brevis* Bb14 | Sourced from ATCC. Isolated from feces. |
| 2 | ***Lactobacillus rhamnosus***  **ATCC-53103** | **—** | Sourced from ATCC. Isolated from feces. |
| 3 | ***Lacticaseibacillus paracasei***  **ATCC-334** | — | Sourced from ATCC. Isolated from dairy products. |
| 4 | ***Bacteroides fragilis***  **HM-709** | *Bacteroides fragilis* CL07T00C01 | Sourced from BEI Resources. Isolated from adult human feces in Massachusetts, USA. |
| 5 | ***Bacteroides fragilis***  **HM-714** | *Bacteroides fragilis* CL03T12C07 | Sourced from BEI Resources. Isolated from adult human feces in Massachusetts, USA. |
| 6 | ***Bacteroides fragilis***  **HM-710** | *Bacteroides fragilis* CL07T12C05 | Sourced from BEI Resources. Isolated from adult human feces in Massachusetts, USA. |
| 7 | ***Bifidobacterium adolescentis***  **HM-633** | *Bifidobacterium adolescentis* L2-32 | Sourced from BEI Resources. Isolated from fecal sample of two-year-old infant in Scotland, United Kingdom. |
| 8 | ***Bifidobacterium breve* HM-411** | *Bifidobacterium breve* EX336960VC18 | Sourced from BEI Resources.  Isolated from mid-vaginal wall in Richmond, Virginia, USA. |
| 9 | ***Bifidobacterium breve***  **HM-1120** | *Bifidobacterium breve*  JCP7499 | Sourced from BEI Resources.  Isolated from female in St. Louis, Missouri, USA. |

**ATCC**: The American Type Culture Collection

**BEI Resources:** The Biodefense and Emerging Infections Resources Repository

**S4 Table. Chemical structures of novel hits from MCE antiviral library**

| No. | Compound ID | Structure |
| --- | --- | --- |
| 1 | Rottlerin | 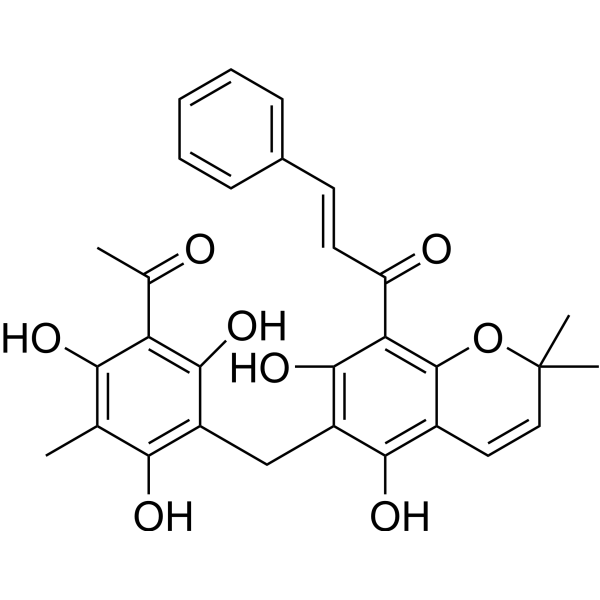 |
| 2 | Dryocrassin ABBA | 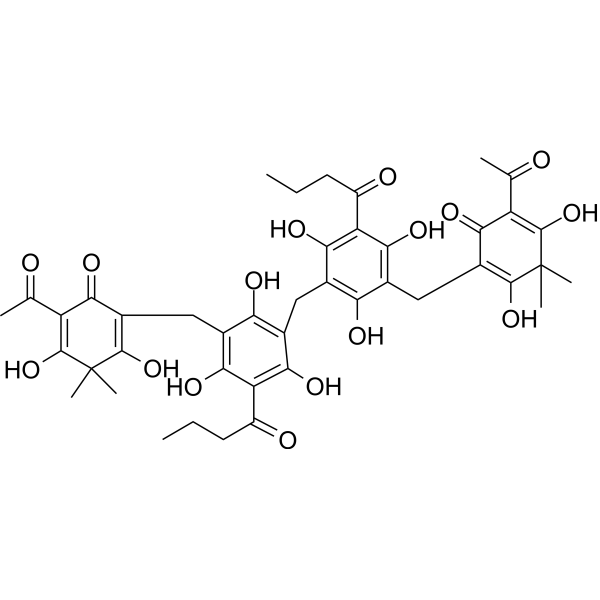 |
| 3 | α-Mangostin | 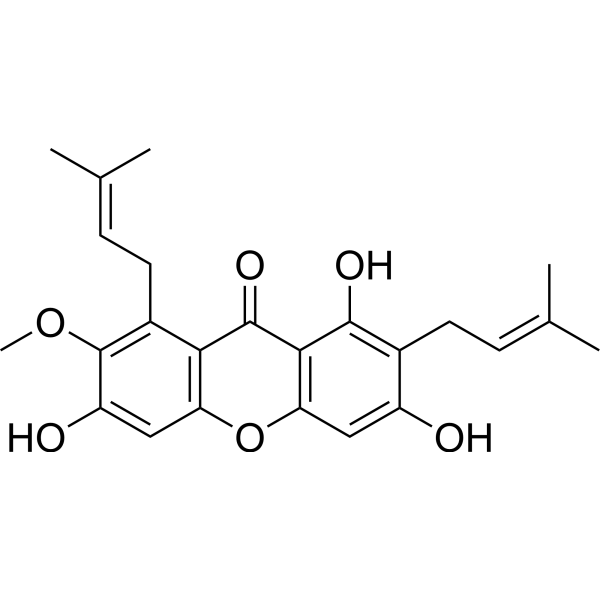 |
| 4 | Obefazimod | 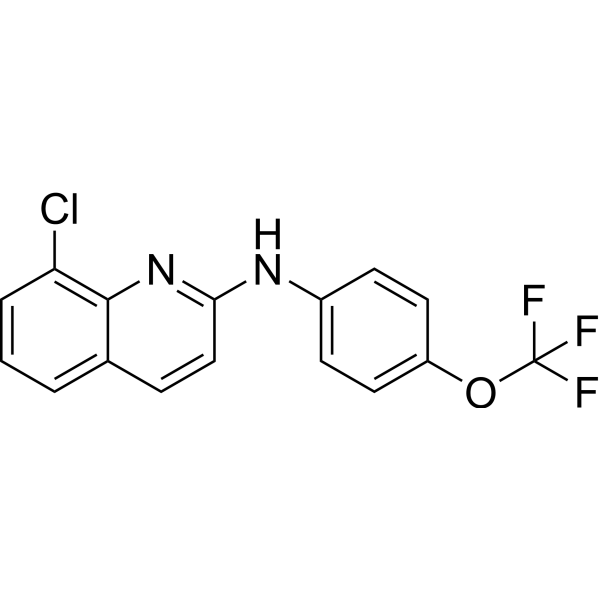 |
| 5 | Baloxavir | 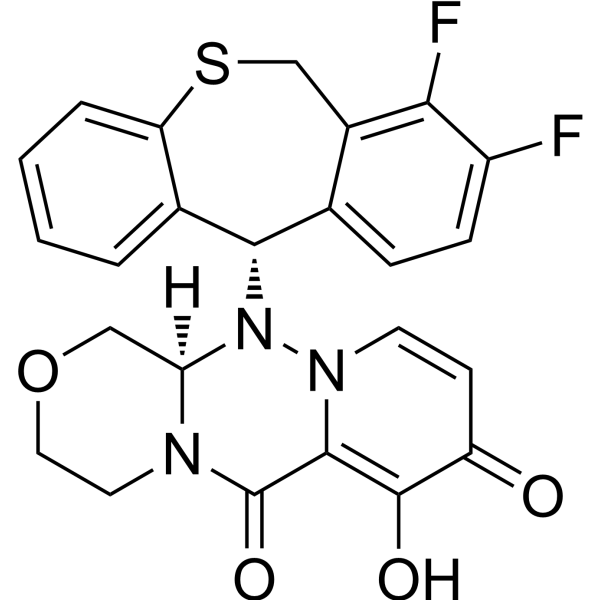 |
| 6 | 4'-O-Methylbavachalcone | 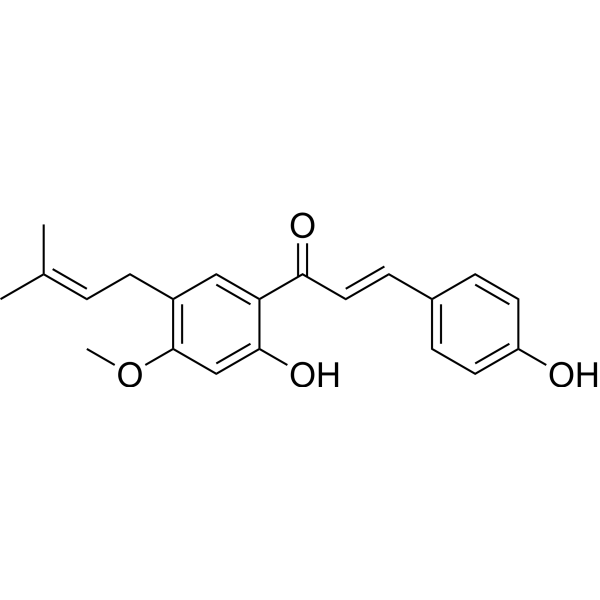 |
| 7 | TMC647055 (choline salt) | 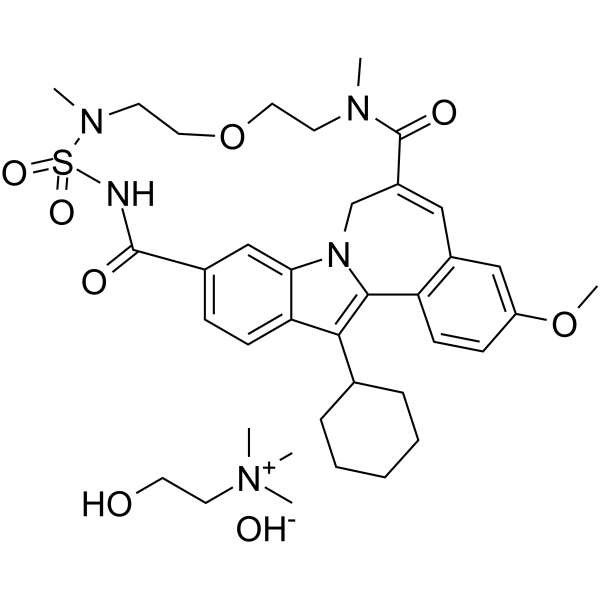 |
| 8 | Brefeldin A | 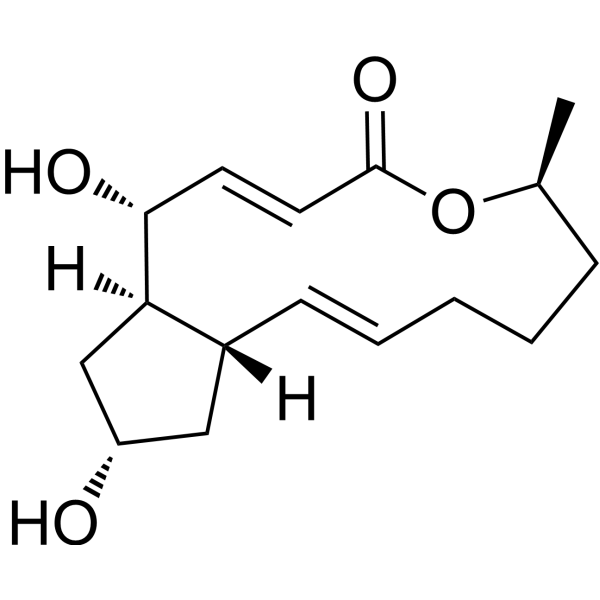 |
| 9 | Maslinic acid | 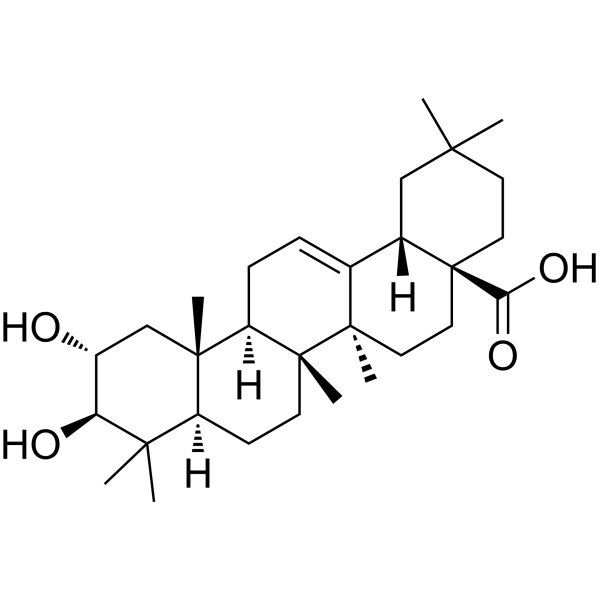 |
